# A weakly supervised deep learning approach for label-free imaging flow-cytometry-based blood diagnostics

**DOI:** 10.1016/j.crmeth.2021.100094

**Published:** 2021-10-25

**Authors:** Corin F. Otesteanu, Martina Ugrinic, Gregor Holzner, Yun-Tsan Chang, Christina Fassnacht, Emmanuella Guenova, Stavros Stavrakis, Andrew deMello, Manfred Claassen

**Affiliations:** 1 Institute for Molecular Systems Biology, ETH Zurich, Zurich, Switzerland; 2 Institute for Chemical and Bioengineering, ETH Zurich, Zurich, Switzerland; 3 Department of Dermatology, University Hospital Zurich and Faculty of Medicine, University of Zurich, Zurich, Switzerland; 4 Department of Dermatology, Lausanne University Hospital (CHUV) and Faculty of Biology and Medicine, University of Lausanne, Lausanne, Switzerland; 5 Internal Medicine I, University Hospital Tübingen, Faculty of Medicine, University of Tübingen, Tübingen, Germany

**Keywords:** deep learning, machine learning, weakly supervised learning, image flow cytometry, cancer cell imaging, high-throughput imaging, peripheral blood mononuclear samples, Sézary syndrome

## Abstract

The application of machine learning approaches to imaging flow cytometry (IFC) data has the potential to transform the diagnosis of hematological diseases. However, the need for manually labeled single-cell images for machine learning model training has severely limited its clinical application. To address this, we present iCellCnn, a weakly supervised deep learning approach for label-free IFC-based blood diagnostics. We demonstrate the capability of iCellCnn to achieve diagnosis of Sézary syndrome (SS) from patient samples on the basis of bright-field IFC images of T cells obtained after fluorescence-activated cell sorting of human peripheral blood mononuclear cell specimens. With a sample size of four healthy donors and five SS patients, iCellCnn achieved a 100% classification accuracy. As iCellCnn is not restricted to the diagnosis of SS, we expect such weakly supervised approaches to tap the diagnostic potential of IFC by providing automatic data-driven diagnosis of diseases with so-far unknown morphological manifestations.

## Highlights

• iCellCnn is a weakly supervised DL approach for label-free IFC-based diagnostics
• The approach does not require training on manually annotated single-cell images
• iCellCnn does not need any prior knowledge of disease-specific morphology
• Demonstration of PBMC T cell morphology-based diagnosis of Sézary syndrome

## Motivation

Imaging flow cytometry has emerged as a powerful tool for high-throughput single-cell morphology analysis and, in conjunction with machine learning approaches, has the potential to transform the diagnosis of hematological diseases. The requirement for manually labeled single-cell images for machine learning model training has so far severely limited the scope and application of such diagnosis strategies. To address this gap, here we present iCellCnn, a weakly supervised deep learning approach for label-free IFC-based blood diagnostics.

## Graphical abstract

**Figure:**
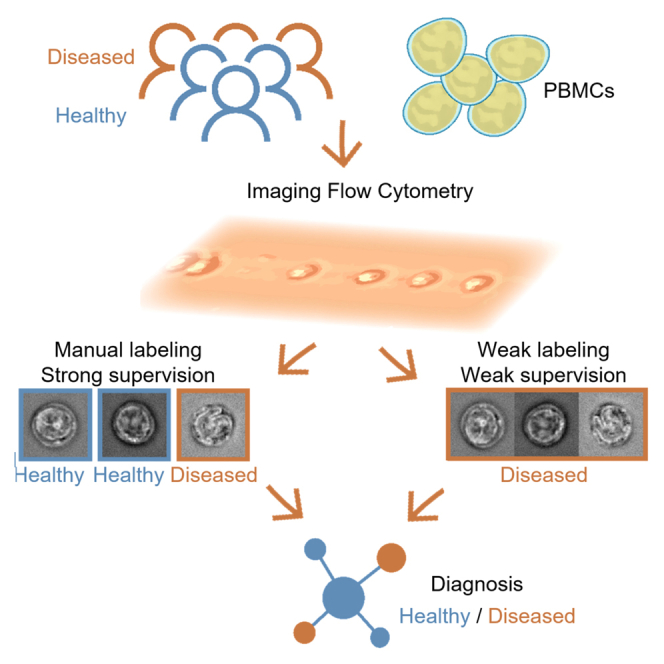

## Teaser

The requirement of manually labeled single-cell images for training of machine learning models severely limits their application in clinical diagnosis strategies. Here, Otesteanu et al. present iCellCnn, a weakly supervised deep learning approach for label-free imaging flow cytometry-based blood diagnostics and demonstrates its utility in morphology-based diagnosis of Sézary syndrome.

## Introduction

The accurate and sensitive diagnosis of pathologies is an essential determinant of patient treatment outcome and prognosis. Given that cell morphology, structure, and chemical composition are linked to physiological function, they can be used as essential markers for diagnosis (Alizadeh et al., 2020). Among such markers, morphology is still the most important criterion for diagnosis and also constitutes a more resource-effective alternative to molecular diagnosis approaches if two conditions are met: (1) morphological patterns are available for accurate and sensitive diagnosis and (2) these patterns can be efficiently and (ideally) automatically evaluated (Bain, 2005; Ford, 2013).

Imaging flow cytometry (IFC) has emerged as a powerful tool for high-throughput single-cell morphology analysis and, in conjunction with machine learning approaches, has the potential to transform diagnosis of hematological diseases (Doan et al., 2018). Traditionally, such diagnostic procedures rely on manual expert microscopical evaluation of blood cell morphology and suffer from subjectivity, limited throughput, and low sensitivity. This situation has motivated an ongoing transition toward molecular diagnostic assays, and shifted the challenge toward identifying suitable molecular targets (Scarisbrick et al., 2018). To circumvent the problems related to the requirement for labels when identifying molecular diagnostic markers, IFC can provide high-resolution morphological information of individual cells at high throughput and thus sensitively identify pathological aberrations of cellular morphology.

Although conventional (fluorescence-based) IFCs allow for relatively high-throughput quantitation of cellular populations, they are costly, mechanically complex, consume large sample and reagent volumes (due to the use of sheath flows to hydrodynamically focus samples into a narrow stream), and require trained personnel for both operation and maintenance (Basiji et al., 2007). In this respect, the development of image-based analysis within microfluidic formats provides an opportunity to develop new platforms for characterizing single cells, which leverage and combine the high-throughput nature of microscale flow cytometry with the enhanced sensitivity of a microscope (Rane et al., 2017; Stavrakis et al., 2019).

Recently, machine learning approaches have been used to classify cellular morphology from IFC data (Hennig et al., 2017). In a recent study, a commercial imaging flow cytometer (Amnis ImageStreamX Mk II, Luminex) operating in a label-free detection mode (bright field and dark field) was used to identify phases in the cell cycle (Eulenberg et al., 2017) and classify white blood cell type (Lippeveld et al., 2020; Nassar et al., 2019). Similarly, the same imaging flow cytometer was used for acute lymphoblastic leukemia diagnostics, with 88% accuracy when using a residual convolutional neural network (CNN) architecture (Doan et al., 2020a). Other studies have focused on integrating imaging technology with deep learning technology. For example, a time-stretch phase-imaging system was used to obtain quantitative phase and intensity images in real time, with feature extraction and deep learning algorithms used to achieve label-free classification of cancerous cells (Chen et al., 2016). An improved version of this imaging platform, termed optofluidic time-stretch microscopy, allowed for ultra-fast acquisition (250,000 frames/s) of bright-field images as well as integrating a deep convolutional autoencoder to identify drug-induced morphological changes in leukemic cells (Kobayashi et al., 2019). Other researchers have also used similar optical approaches in conjunction with deep learning to realize morphology-based identification and enumeration of aggregated platelets in blood (Jiang et al., 2017). However, these approaches possess significant limitations that preclude their diagnostic use in clinical applications. Most importantly, all of the above approaches incorporate strongly supervised machine learning models, whose establishment requires difficult-to-obtain examples of manually annotated single-cell images, ideally in large numbers. This requirement further precludes their application to disease entities without *a priori* knowledge of diagnostic morphological patterns.

Herein, we present iCellCnn, a weakly supervised deep learning approach for label-free IFC-based clinical diagnostics that circumvents the necessity for manually annotated single-cell images. We demonstrate iCellCnn’s capabilities for diagnosis through the diagnosis of Sézary syndrome (SS) (Broder et al., 1976), an aggressive form of cutaneous T cell lymphoma (Bobrowicz et al., 2020; Phan et al., 2016; Willemze et al., 2019). SS is characterized by circulating tumor T cells with cerebriform nuclei that serve as potentially useful morphological diagnostic features. This morphological manifestation of malignant T cells with cerebriform nuclei consisting of overlapping folds and clefts (Lutzner et al., 1971) offers a unique opportunity to visualize these cells within a patient’s blood. Accurate detection of these cells could contribute to diagnosing the disease at an early stage, which is of high importance for timely and effective treatment.

## Results

### iCellCnn: Weakly supervised learning of diagnostic cellular morphology from IFC data

**Figure 1:**
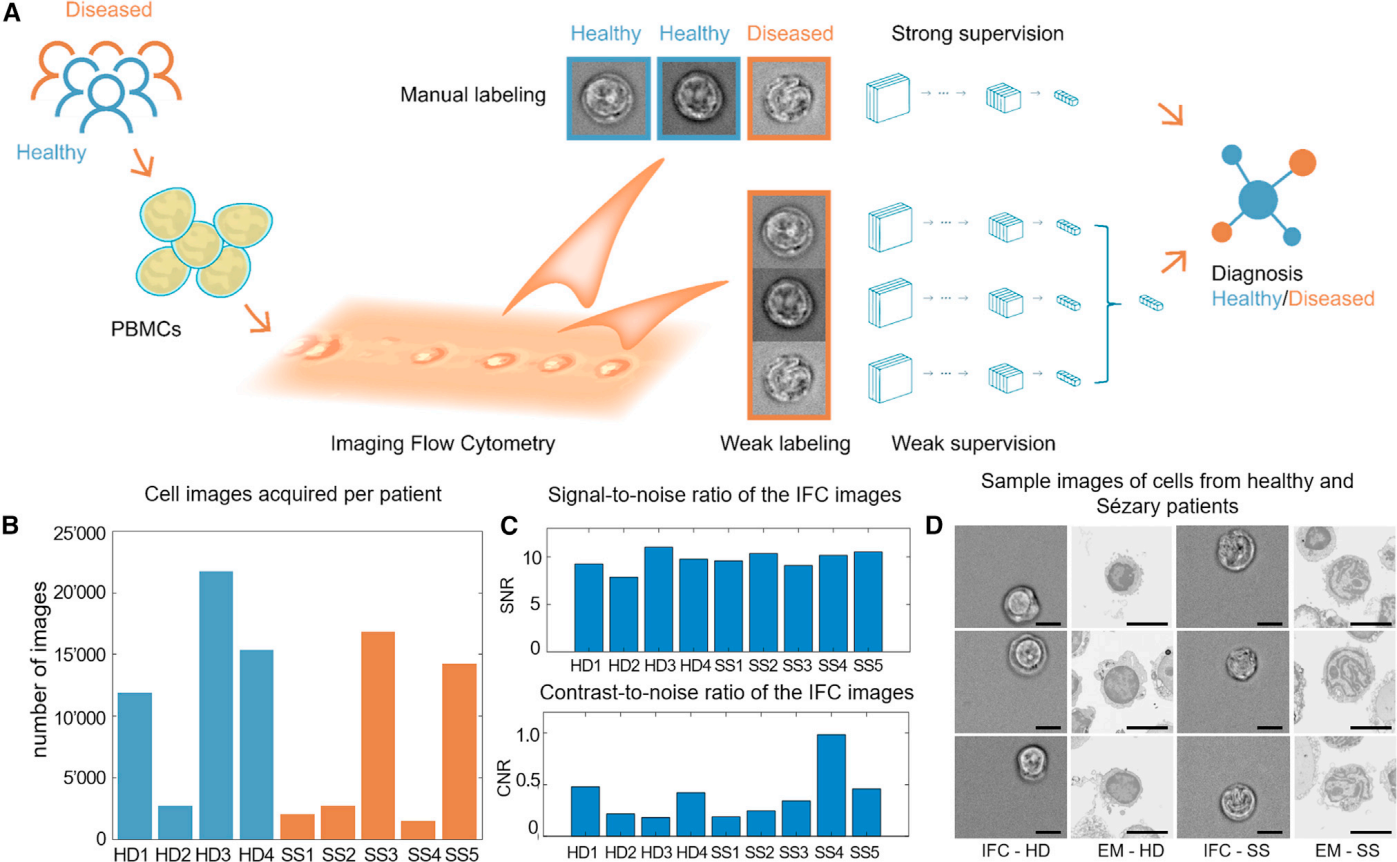
Workflow for label-free IFC-based clinical diagnostics (A) Peripheral blood mononuclear cell samples are imaged using our IFC platform. The digitized cell images are preprocessed and then used to train a machine learning model, with labels either at a whole-sample level or (weak) labels at an individual image level. Multiple images are pooled together in a bag of cells, and used to train a classifier to provide a final diagnosis probability. (B) Number of cell images acquired per patient sample. (C and D) (C) Signal-to-noise ratio and contrast-to-noise ratio of the IFC images. (D) Example images of healthy donor cells and Sézary syndrome patient cells captured using both imaging flow cytometry and high-resolution electron microscopy. Scale bars, 5 μm.

iCellCnn is a weakly supervised approach for classifying a patient's disease status on the basis of IFC data from a clinical specimen, i.e., an image collection of cells that are specific as well as unspecific to the disease (Figure 1A). In contrast to conventional strongly supervised approaches, which are tedious and whose establishment requires individual cell images labeled as “specific” or “unspecific” to the disease, iCellCnn can be trained by using only the information on the disease state at the level of the specimen, i.e., the entire cell image collection that results from the specimen. iCellCnn circumvents the requirement for strong supervision by using a set of images rather than individual images as an input, in a similar fashion as reported for conventional (non-imaging) flow cytometry data (Arvaniti and Claassen, 2017). In contrast to the comparably low dimensionality of the conventional flow cytometry input (Arvaniti and Claassen, 2017), our approach employs high-dimensional IFC single-cell images summarized by a vector of relevant morphology features defined in a data-driven fashion (Goodfellow et al., 2020). Specifically, iCellCnn utilizes a deep convolutional autoencoder architecture (Goodfellow et al., 2020) to represent each image as a feature vector in a latent space representation.

Multiple cell image representations from the same patient specimen were concatenated as a “bag of cells” (BoC) in a two-dimensional feature vector. Here, the most relevant features were learned and enhanced by mean pooling, with the resulting one-dimensional feature vector used as an input to a random forest classifier, indicating the presence or absence of diseased cells in the input cell collection. Training of the iCellCnn model defines, in a data-driven fashion, morphological patterns of disease-specific cells, while the model learns to ignore confounding non-disease-specific cells, and ultimately enables diagnosis of diseased patients from the IFC measurements.

### IFC of peripheral blood mononuclear cells of SS patients

Peripheral blood mononuclear cell (PBMC) samples were collected from four healthy donors (HDs) and five SS patients. In some cases, fluorescence-activated cell sorting was performed to enrich the data pool for SS T cells (see STAR Methods), as these cells are a subset of immune cells contained in the PBMC samples.

The resulting cell suspensions are then introduced into the microfluidic device and elasto-inertially focused into a single file. An in-house developed, small-footprint, cost-effective IFC that incorporates a microfluidic platform for three-dimensional cell focusing was used for the acquisition of individual PBMC images at high throughput (see STAR Methods; Figure S1). Here, cells are elasto-inertially focused if the channel dimensions are adjusted to yield a blockage ratio, *b* (*b* = *a*/*h*, where *a* is the average cell diameter, and *h* is the channel diameter), smaller than 0.25 (Romeo et al., 2013). A straight microchannel with a cross-section of 45 × 45 μm in combination with a 1,000 ppm, 1 MDa PEO solution was used to focus cells in all experiments. More than 100,000 images were collected in total, with at least 2,000 cell images per patient as shown in Figure 1B. An embedded graphics processing unit (GPU) platform was used to pre-filter and save only “in focus” images containing cells within the field of view (see STAR Methods; Figure S1C). Given the relatively shallow depth of field of the 60× imaging objective, obtaining high-resolution images of flowing cells necessitates focusing the individual cell into a single file within the working distance of the lens. Figure 1D highlights the image quality achieved by using such an approach, with a mean signal-to-noise ratio (SNR) of 9.8 (SD 1.4) and a mean contrast-to-noise (CNR) of 0.45 (SD 0.59) when imaging cells at a flow rate of 55 μL/min (Figure 1C; see STAR Methods for the calculation of SNR and CNR). Representative IFC images highlight the typical irregular nucleus, containing lobulations and indentations, of SS cells (Figure 1D), as observed in the associated high-resolution scanning electron microscope (SEM) images (see STAR Methods for SEM acquisition and protocol details). The recorded images are used to train the weakly supervised iCellCnn for the identification of disease-specific morphological signatures.

### Identification of a diagnostic cellular morphology in PBMCs of SS patients with iCellCnn

**Figure 2:**
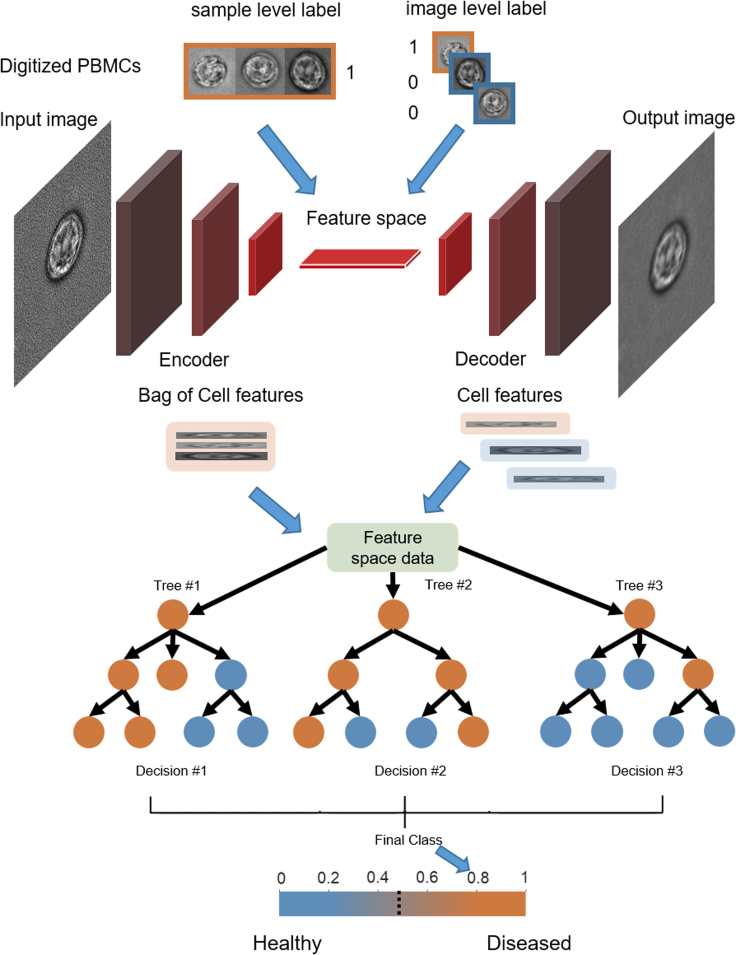
iCellCnn model architecture The digitized cell images are preprocessed and used to train a machine learning model, with labels either at an individual image level or (weak) labels at the whole-sample level. A convolutional autoencoder is trained as a feature extractor to represent each single image in a latent space. Multiple images are pooled together in a bag of cells, which is represented in a latent space (bag of cell features), and used to train a random forest classifier to provide the final diagnosis probability.

**Figure 3:**
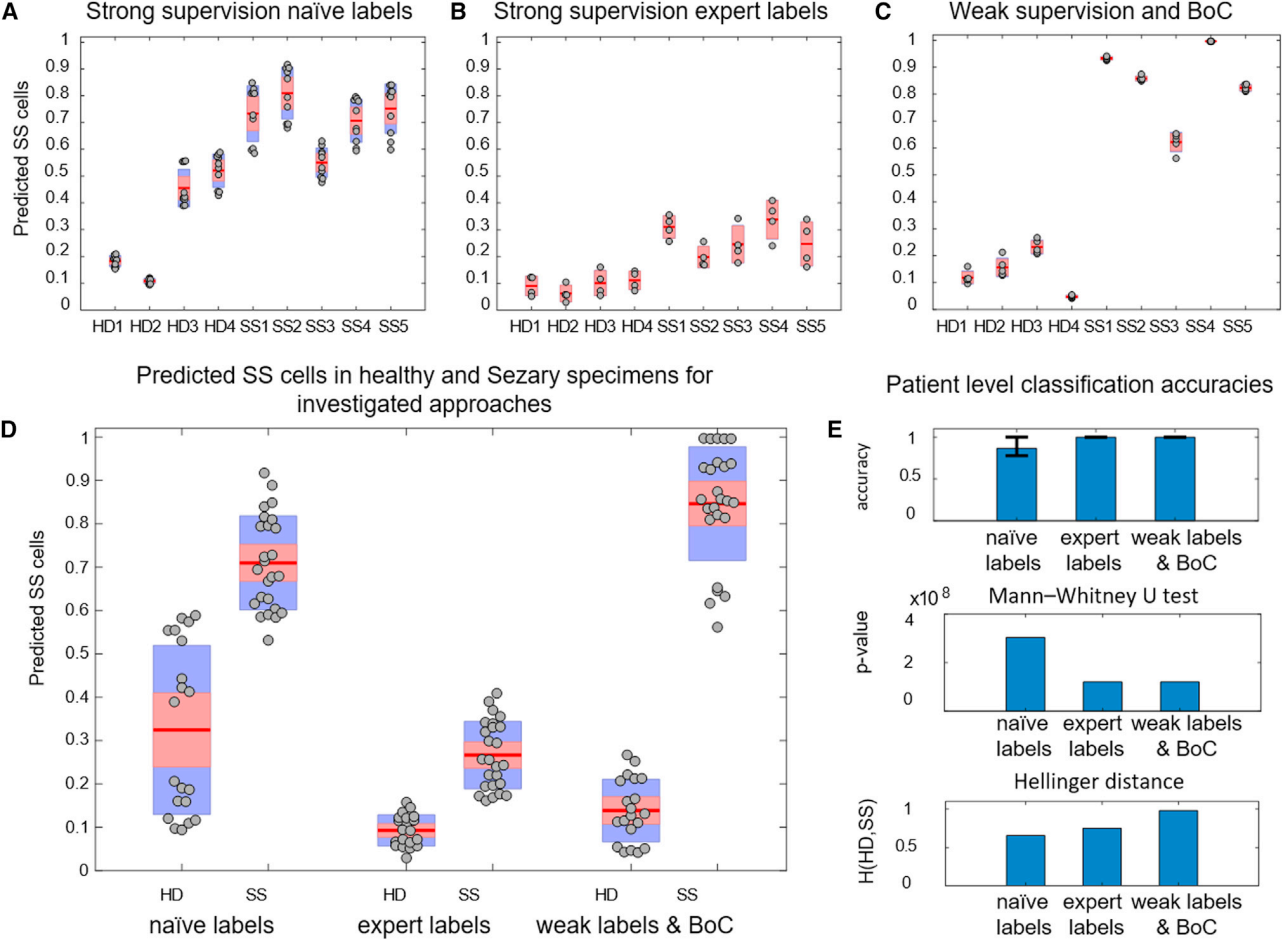
Predicted percentage of cells with atypical (Sézary) morphology in the blood of healthy and diseased specimens This was achieved using (A) strong supervision using naive labels, (B) strong supervision with a subset of annotations on diseased cells, (C) bag of cells approach using weak supervision, and (D) group-wise amounts of healthy donor and Sézary syndrome patient predicted cells with atypical (Sézary) morphology. (E) Patient-level classification accuracy, p values, and Hellinger distance between healthy and diseased probability distributions. The error-bars represent the test accuracy standard deviation across five training experiments.

We assessed iCellCnn's (see Figure 2) capability for diagnosis of SS and compared it with strongly supervised learning approaches. Specifically, we considered two variants of strong supervision, naive and manual image annotation, where for both variants the label of each individual cell image was used when training the model. In the *naive annotation* approach we implemented a CNN model based on the ResNet18 architecture (He et al., 2016), consisting of 18 convolutional layers, followed by a fully connected layer and a softmax activation function. The model was trained on cell images with individual labels. We defined a naive annotation, where all the cells from the same patient specimen were assigned with the respective patient’s health status (i.e., healthy or diseased). The model was trained on the basis that SS patients have a larger percentage of morphologically atypical cells. Throughout this work, when reporting the classification results and number of diseased (Sézary) cells in the blood of HDs and SS patients, this is read as the number of cells with atypical morphology as assigned by the trained model. A leave-one-out-cross-validation (LOOCV) approach was used for training and evaluating the performance of the model. All the performance results are reported on the validation set (see STAR Methods). A patient-wise breakdown of the predictions of our approach can be seen in Figures 3A–3C and S2B, with the healthy donors denoted as HD1–4, and the SS patients as SS1–5. Using this approach, the healthy specimens contained a mean of 31.6% (SD 18.3%) of their cells classified as diseased and the SS patients a mean of 71% (SD 12.1%), with a patient-level classification accuracy of 86.7% (SD 9.3%) (Figure 3A).

In the *manual* (*expert labels*) *annotation* approach, the same model was used and trained on naively annotated cells of healthy individuals and a subset of manually annotated pathological cells from SS patients. A total of 1,000 cell images were annotated as pathological, on the basis of the clear cerebriform appearance of their nuclei. The model was trained by using only cell images from the healthy specimen data and the manually annotated pathological cell images in an LOOCV approach. Using such an approach, healthy specimens had a mean of 9.1% (SD 3.9%) of their cells classified as diseased and SS a value of 26.8% (SD 7.7%) (Figure 3B). The model accuracy was also evaluated on the expert annotated Sézary cell images, where it achieved a mean accuracy of 94.2% (SD 2.7%) (Figure S2C). A class separating threshold that maximizes the distance between the two classes was determined for the predicted SS cell frequency. A threshold value of 15.94% that led to a patient-level classification accuracy of 100% was computed in an LOOCV approach as the mean between the highest predicted SS cell frequency from the healthy donor class and the smallest prediction from the diseased class.

For the *weak supervision approach* (iCellCnn), a convolutional autoencoder with five convolutional layers in the encoder and decoder blocks was implemented to extract features from all specimens in an unsupervised manner. Multiple images from the same patient were then grouped in a BoC, and assigned collectively a (weak) label corresponding to the disease state of the respective patient.

After representing each cell image as a feature vector computed by the encoder block of the convolutional autoencoder, BoCs of the patients could be represented as “bags of cell features” (BoCF). The BoCF was then used to train a random forest classifier. Figure 2 illustrates a flow-chart of this approach. Using this method, all patients were correctly classified, with the healthy specimens having a mean of 13.9% (SD 7.2%) of their cells classified as diseased and the SS patients being assigned a mean of 84.6% (SD 13.1%) of their cells classified as diseased (Figure 3C). Comparison of the approaches across the complete patient cohort demonstrates the excellent separation capability of iCellCnn (Figures 3D and 3E). The Mann-Whitney U test detected significant differences between the mean estimated cell frequency of the two classes for all approaches.

Although strong supervision using expert labels and weak supervision approaches achieved similar patient-level classification accuracies (Figure 3E top) and p values (Figure 3E middle), the weak supervision approach achieved superior separation between classes, with a Hellinger distance of 0.98, compared with strong supervision approaches using expert labels (0.75) and naive labels (0.66) (Figure 3E bottom; see STAR Methods). The Hellinger distance metric was used to quantify the similarity between the classes, with a score of 1 signifying a maximum separation of the classes. On the basis of an analysis of the aforementioned results, the weak supervision approach, iCellCnn, achieved considerable improvement on disease predictions compared with those achieved with strong supervision models, without the need of manually providing annotated labels and the overhead associated with this process.

## Discussion

We present iCellCnn, a weakly supervised approach for the disease diagnosis from patient blood samples by using image flow cytometry. To the best of our knowledge, this is the first study that implements a combination of label-free IFC and machine learning in a weakly supervised manner for disease diagnosis, i.e., a data-driven approach circumventing the limiting requirement of single-cell image annotations. Furthermore, this is the first study using machine learning morphology-based diagnosis of SS.

In the current study, we developed a simple yet high-performance imaging flow cytometer with dimensions of 20 × 25 × 30 cm (weighing 7.5 kg). Compared with existing IFC platforms, the presented instrument is significantly cheaper and more compact, with the system costing less than 10,000 Swiss Francs to build. This cytometer comprises a straight microfluidic channel and does not require hydrodynamic focusing of the sample; a feature that makes the system robust and simple to operate. In addition, the platform has a simple hardware configuration, only incorporating a custom optical unit for bright-field imaging, a moderate numerical aperture objective lens, a low-cost CMOS camera, and a single-channel microfluidic chip. In this regard, it is important to note that, even with moderate bright-field resolution, we are able to demonstrate the predictive power of the iCellCnn CNN in morphological classification of Sézary cells. The presented pipeline is capable of enumerating single-cell images in flow and identifying morphological features of the acquired images by using minimal hardware and, in conjunction with the iCellCnn approach, to learn diagnostic cellular morphology. By virtue of the technical simplicity of the device and the portability of the machine learning analysis, we expect that this integrated IFC approach can be established in a plethora of clinical laboratories.

First, we presented a strong supervision approach with naive labels, where 50% of HDs and 80% of SS patients were correctly classified, considering a 50% boundary (prediction) threshold and a 95% confidence interval. Although the 70% mean of identified Sézary cells in the SS patients might seem significantly larger than the healthy donor mean of 31%, two of the healthy donors (HD3 and HD4) had means closer to the SS patients than to their healthy counterparts. Moreover, these predicted values are larger than those described in the literature (Bernengo et al., 2001; Meyer et al., 1977). Therefore, we hypothesize that the larger number of identified diseased cells does not reflect the actual number of cells with Sézary morphology, but rather the fact our naive annotation model was unable to generalize precisely to other data on the basis of the naive labels provided, as well as to a lower extent for the manual expert labels.

To improve the classification accuracy, we then used a similar approach after manually annotating a subset of SS patient cell images as the diseased state. This improved the model predictions, with all the HD individuals having less than 16% of their cells counted as morphologically atypical (2.9%–15.8%), and all the SS individuals having more than 16% of their cells counted as morphologically atypical (16.1%–40.9%). Although the separation between the two classes was improved, the total number of predicted diseased cells for the SS patients was low. This can be explained by the fact that only a subset of the data were manually annotated and, in addition, because of the time constraints of this process, we only annotated the positive (diseased) samples. This setup consequently results in morphologically abnormal T cells from healthy individuals being labeled as “healthy,” therefore distorting the model predictions. These issues indicate the limitations that accompany automatic diagnosis approaches depending on manual annotation of single-cell images.

We assume that the trend toward inflated Sézary annotations is related to the observation that a fraction of Sézary cells are morphologically indistinguishable from normal lymphocytes. As stated in (Bernengo et al., 2001), the percentage of Sézary cells with abnormal morphology can range from 8% to 90%, with a mean of 32.8% (SD 23). Cells with atypical morphology can also be found in healthy individuals (Meyer et al., 1977) at between 3.2% and 13.3%, with a mean of 8.7% (SD 3.5). It is worth noting that these studies were conducted by using high-resolution electron microscopy, as opposed to lower-resolution bright-field imaging. Nevertheless, there was an overlap between the classes, with four out of six HDs having their numbers within the range of SS patients. Interestingly, in our study, when using the manual expert label strong supervision approach, similar values were identified for healthy (2.9%–15.8%, mean of 9.1%) and diseased patients (16.1%–40.9%, mean of 26.8%), albeit with a narrow separation between classes of 0.3%.

To overcome such limitations, we propose iCellCnn as a weakly supervised model. Because of the possibility that healthy individual PBMC samples might consist of lymphocytes with morphological patterns similar to Sézary cells, and SS patient PBMC samples might comprise non-pathological cells, our weakly supervised model is based on a BoC approach that pools cell images from the same patient and processes them under a collective label. In addition, the use of a convolutional autoencoder offered the advantage that relevant morphological features can be compactly extracted in an unsupervised manner, which presumably increases feature (the learned latent space representations) robustness to noise (Vincent et al., 2008) in the cell images, given that it does not use any prior assumptions regarding the data (Carbonneau et al., 2018). A random forest was chosen as the final classification layer because it requires considerably less data for training when compared with conventional neural networks. This is essential, because by using the BoC approach the amount of data (bags) for training is reduced by the same factor as the size (number of cells) of the bag. This approach achieved a 100% patient-level classification accuracy, with all healthy individuals being assigned a lower frequency (<27%) and all the SS patients being assigned a higher frequency of their cells counted as diseased (>56%).

Given that our model estimates the frequency of occurrence of cells with Sézary morphology, we believe that our approach would be useful in examining disease progression, assuming that a patient with a more advanced diseased state would exhibit more cells with Sézary morphology, and therefore our model would yield a higher SS cell frequency.

We speculate about the degree of potential improvement of classification performance. Accordingly, we estimated the upper bounds of classification accuracy and separation by using the following heuristic. We implemented a BoC model where the autoencoder was not trained on all cells as before, but instead only on manually annotated diseased cells (Figures S2A and S2B). The resulting latent representation of the IFC images would therefore be specific for features of diseased cells only, and not for the spectrum of features characterizing all cells. In this approach, healthy patients had less than 22% of their cells counted as diseased, compared with more than 61% for SS patients. Our weak supervision approach achieved similar classification performance to that of the strong supervision model in terms of the number of cells classified as positive (for the healthy and diseased specimens), without the need to provide manually annotated labels. Such a capability avoids several limitations. First, annotating data of certain pathologies requires expert domain knowledge of a physician. Second, the process of labeling a large amount of data are time-consuming and laborious and can lead to labeling errors caused by human intervention. Furthermore, there are many diseases without *a priori* known diagnostic cellular morphology. Thus, because cell images in these situations cannot be manually annotated, conventional strong supervision approaches are not applicable for training automatic diagnosis models.

Although the specimens investigated in this study were either HDs or patients diagnosed with SS—providing a clear label for the dataset, the diagnosis label of the patients was not based on cell morphology, but based on molecular diagnostic assays and patient symptoms. The phenotype between the healthy and diseased cells evaluated by our machine learning model was made by using donor and patient cell images, therefore resulting in a more complex and challenging dataset. This is because a fraction of Sézary cells are morphologically indistinguishable from normal lymphocytes (Bernengo et al., 2001), whereas cells with atypical morphology can also be found in healthy individuals (Meyer et al., 1977).

In our study, using the IFC bright-field images, a total number of 1,000 cells were annotated as pathological, on the basis of the clear distinct cerebriform appearance of their nuclei, and accounting for 1.1% of the total number of SS patient cell images. On the basis of the relatively low percentage of cells with distinct cribriform morphology, we believe our method would achieve similar results on other datasets where the diseased cell frequencies are comparably low.

Digital hematology applications based on machine-learning-assisted IFC have great potential to achieve more accurate and significantly faster classification with minimal human intervention. By integrating IFC and deep learning, our iCellCnn approach has been shown to successfully provide morphology-based diagnosis of SS. Currently, commercially available IFC systems require complex equipment and operation by trained personnel, which prevents their deployment in almost all field or clinical environments. These drawbacks can be mitigated through the adoption of our pipeline that incorporates portable, simple, and high-performance IFC combined with a weakly supervised approach. Although the focus of this study was on SS, our approach is almost certainly applicable to a variety of other hematological malignancies or other diseases inducing morphological changes in the blood cell compartment, such as leukemia, or even inflammatory skin diseases. Given that most adults undergo routine blood tests every 1 to 5 years (Boulware et al., 2007) our proposed pipeline can lead to routine screening of a wide range of pathologies, such as cutaneous lymphomas and non-neoplastic disease, which traditionally are considered infeasible due to cost reasons.

### Limitations of the study

All the data in our study were acquired by using an in-house built IFC platform. We assume that refining the model for data acquired from a different IFC might involve re-training of the iCellCnn. Given that data availability of the same pathology might be limited, an alternative approach could involve using domain adaptation (Tomczak et al., 2020), where an image translation model is learned between the source (in-house IFC device) and the target domain (new IFC device).

A limitation of our weakly supervised approach is that the feature extraction network of iCellCnn was decoupled from the BoC classification layer. Other weakly supervised approaches based on CNNs (Chikontwe et al., 2020; Sudharshan et al., 2019) or Multi-layer Perceptron Networks (Wang et al., 2018) have been proposed. Doan et al. (2020b) used another weakly supervised approach for assessing the quality of stored red blood cells, using CNN as a feature extractor on single-cell images; features that were afterward used to train a one-dimensional UMAP embedding. However, the denomination as “weakly supervised” learning is used in different ways by us and Doan et al., and discussed generally in (Zhou, 2017). Following our notion, Doan et al. use strong supervision in terms of associating a label to each single-cell image and learning a feature extractor to map the two spaces, and weak (or self) supervision for training the UMAP embedding. Our approach associates a label with a BoC, which we refer to as weak labeling of a set of inputs, instead of as strong labeling of individual inputs.

We opted for a different approach, because by using BoCs instead of single images for training a CNN the memory capacity increases linearly with the bag size and memory errors can be encountered. This would limit the number of cell images present in a bag, as well as the batch size (training data used in one iteration) even on a high-performance GPU cluster. Moreover, as with the BoC approach, the number of available BoCs scales inversely with the number of cells in a BoC, leading to suboptimal CNN training due to limited data availability.

It is significant to note that iCellCnn is not restricted to the diagnosis of SS and we expect this weakly supervised approach to be extendable to the diagnosis of other diseases with morphological aberration in blood cells. Although generalization to other diseases is likely to be dependent on the degree of morphological similarity between the new investigated pathology and SS, a transfer learning approach could be used to limit this dependence (Khan et al., 2019). In such an approach, the entire or part of the already trained CNN could be used for feature extraction, and only the final classification layer would have to be re-trained.

## STAR★Methods

### Key resources table

**Table:** 

REAGENT or RESOURCE	SOURCE	IDENTIFIER
**Antibodies**		

anti-human CD3 monoclonal antibody conjugated with PerCP	Miltenyi Biotec, Gladbach, Germany	clone BW264/56, Cat#130-096-910; RRID: AB_2725962

**Biological Samples**		

Sezary Syndrome patient PBMCs	University of Zürich Biobank	Biobank project (EK No. 647)
Healthy donor PBMCs	Blutspende SRK Zürich	N/A

**Chemicals, Peptides, and Recombinant Proteins**		

SYTOX™ Red Dead Cell Stain	ThermoFisher Scientific	Cat#S34859
Formaldehyde solution	Sigma-Aldrich, Buchs, Switzerland	Cat# 47608, CAS: 50-00-0
Poly-L-lysin	Sigma-Aldrich, Buchs, Switzerland	Cat# P6282, CAS: 25988-63-0
Glutaraldehyde solution	Sigma-Aldrich, Buchs, Switzerland	Cat# G7776, CAS:111-30-8
Osmium tetroxide	Sigma-Aldrich, Buchs, Switzerland	Cat# 201030, CAS:20816-12-0
Thiocarbohydrazide	Sigma-Aldrich, Buchs, Switzerland	Cat# 223220, CAS:2231-57-4
Epoxy embedding medium	Sigma-Aldrich, Buchs, Switzerland	Cat# 45345
Uranyl Acetate 98%, ACS Reagent	Polysciences	Ref: 45345

**Deposited Data**		

IFC image data	This paper	https://doi.org/10.5281/zenodo.5391154

**Software and Algorithms**		

python 2.7	Python Software Foundation	https://www.python.org/
tensorflow 1.7	Abadi et al. (2016)	https://www.tensorflow.org/
keras 2.1.5	Chollet (2018)	https://keras.io/
scikit-learn 0.19	Pedregosa et al. (2011)	https://scikit-learn.org/

### Resource availability

#### Lead contact

Further information and requests for resources and reagents should be directed to and will be fulfilled by the Lead Contact, Prof. Manfred Claassen (Manfred.Claassen@med.uni-tuebingen.de).

#### Materials availability

This study did not generate new unique reagents.

### Experimental model and subject details

#### Human blood samples from patients and healthy individuals

We used blood samples from SS patients whose diagnosis was established according to the recommendations of the International Society for Cutaneous Lymphomas (ISCL) and World Health Organization–European Organization of Research and Treatment of Cancer (EORTC). These include clinical presentation with erythroderma and lymphadenopathy and neoplastic T cells as determined by an absolute Sézary cell count of 1000/mL, or an expanded CD4+ T-cell population resulting in a CD4/CD8 ratio >10, CD4+/CD7- cells >30%, or CD4+/CD26- cells >40%, in combination with monoclonal rearrangement of the T cell receptor (Thiers, 2008; Willemze et al., 2019). Blood samples from SS patients were collected from the University of Zürich Biobank. All patients provided consent to use samples and the related clinical data, according to the Biobank project (EK No. 647) and the “Generalkonsent des USZ” of the University Hospital Zürich. Blood from healthy individuals was obtained anonymously from Blutspende SRK Zürich. The study was conducted in accordance with the principles of the Declaration of Helsinki and the design was approved by the Institutional Review Board of the Canton of Zürich (KEK-ZH-Nr. 2018-02326). Blood from five SS patients and four healthy donors was used in this study. Information regarding age, sex or gender identity of the healthy donors could not be provided due to anonymity reasons. The SS patients were three Caucasian elderly male (70, 71, and 78 years old) and two Caucasian elderly female (71, 74 years old).

### Method details

#### Isolation of T cells and FACS

Peripheral blood mononuclear cells (PBMCs) from patients with SS and healthy individuals were obtained via Ficoll/Paque density gradient separation (17-1440-03, GE Healthcare, Opfikon, Switzerland). For FACS experiments, the PBMC samples were stained with anti-human monoclonal antibodies: anti-human CD3 monoclonal antibody conjugated with PerCP (clone BW264/56, #130-096-910, Miltenyi Biotec, Gladbach, Germany), anti-human CD4 monoclonal antibody conjugated with APC-Vio770 (clone REA623, #130-113-223, Miltenyi Biotec,Gladbach, Germany) and anti-human CD8 monoclonal antibody conjugated with PE-Vio770 (clone REA734, #130-110-680, Miltenyi Biotec, Gladbach, Germany). SYTOX™ Red Dead Cell Stain (#S34859, ThermoFisher Scientific, Zurich, Switzerland) was used for dead cell removal. The live CD3+/ CD4+/ CD8- T cells were subsequently sorted on a BD FACSAria Fusion (BD Biosciences, Allschwil, Switzerland) flow cytometer.

#### Sample preparation for IFC

In all experiments, after cell sorting using FACS, cells were fixed with a 4% formaldehyde solution, washed with Dulbecco's Phosphate-Buffered Saline (DPBS, Life Technologies, Zug, Switzerland) and resuspended in a viscoelastic polyethylene oxide solution (PEO) solution to allow for elasto inertial focusing. Multiple parameters must be controlled to ensure efficient focusing cells into a single file. These include the molecular weight of the polymer, concentration of the polymer solution, microfluidic channel geometry, the blockage ratio and flow rate of the suspension. A detailed description of the specific influence of each of these parameters can be found elsewhere (Holzner et al., 2017). Based on this analysis, current experiments were carried out using a 4400 ppm/1 MDa PEO (Sigma-Aldrich, Buchs, Switzerland) solution. A stock solution at a concentration of 10,000 ppm was prepared and aged at room temperature for a month, to ensure stability. The stock solution was diluted with DPBS to the desired concentration and used immediately or stored at 4°C.

#### Microfluidic device fabrication

Microfluidic devices containing a straight microchannel (with a 45x45 μm cross-section) were fabricated using standard soft-lithographic techniques. The two-dimensional channel pattern was designed using AutoCAD (Autodesk, San Rafael, USA) and printed onto a transparent film photomask (Micro Lithography Services Ltd, Chelmsford, United Kingdom). This photomask was subsequently used to pattern an SU-8 (Microchem Corporation, Westborough, USA) coated silicon wafer using conventional photolithography. A 10:1 mixture of polydimethylsiloxane (PDMS) monomer and curing agent (Sylgard 184, Dow Corning, Midland, USA) was poured over the master-mold and peeled off after polymerization at 70°C for 4 hours. Inlet and outlet ports were punched using a holepuncher (Technical Innovations, West Palm Beach, USA) and the PDMS substrate subsequently bonded to a planar glass substrate with a thickness of 170 μm (Menzel-Glaser, Braunschweig, Germany), after treating both surfaces with an oxygen plasma (EMITECH K1000X, Quorum Technologies, East Sussex, United Kingdom) for 60 seconds. After bonding, microfluidic devices were maintained at 70°C for at least 4 hours to recover hydrophobicity and prevent adhesion of the cells to the channel walls.

#### Device operation

The cell suspension was loaded into a 1 mL syringe (Gastight Syringes, Hamilton Laboratory Products, NV, USA) and delivered at flow rates of up to 1.5 μL/min using a precision Aladdin syringe pump (World Precision Instruments, Friedberg, Germany). Settling of cells within syringes was minimized by matching the density of the medium to the cell suspension using a 36% v/v Optiprep Density Gradient Medium (Sigma-Aldrich, Buchs, Switzerland).

#### IFC system

The IFC system comprised a homemade microscope and a USB 3 CMOS camera with a pixel size of 5.86 μm (UI-3060CP Rev. 2, IDS Imaging Development Systems, Obersulm, Germany). Illumination of the microscope was provided by a green light-emitting diode (LED) (CBT-120, Luminus, Sunnyvale, USA). LED light was coupled into a liquid waveguide (LLG05-4H, Thorlabs, Dachau, Germany) using a set of two lenses (ACL5040U, Thorlabs, Dachau, Germany) in a 4f configuration. Light coming from the waveguide was focused to a tight spot in the imaging plane of the microfluidic channel using two lenses (OSL2FOC, Thorlabs, Dachau, Germany). A 60x 0.70 NA objective (Nikon, Plan Fluor, ELWD, Nikon, Zurich, Switzerland) mounted on a *z*-movable stage (SM1Z, Thorlabs, Dachau, Germany) was used to collect brightfield images. A convex lens with a focal length of 200 mm (LA1708-A 1" 200.0 mm, Plano Convex Lens, Thorlabs, Dachau, Germany), mounted in a 30 mm optical cage system (Thorlabs, Dachau, Germany) was used as the tube lens and a dielectric mirror (KCB1C, Thorlabs, Dachau, Germany) guided the light to the USB 3 CMOS camera. In addition to these components, the system incorporates an embedded computing platform for real-time image processing based on machine learning. The embedded GPU platform was used to pre-filter and save only relevant images for a classification task (i.e. in focus images with cells in the field-of-view). Cell images outside the field-of-view, images containing cell debris and empty images were discarded. Figure S1C presents an overview of typical images acquired prior to filtering. Pre-filtering was performed using a convolutional neural network architecture, with three sequences of convolutional and max pooling layers, followed by two fully connected layers. In order to pre-filter images which were not relevant, a total of 2253 empty images were collected by the IFC apparatus, without providing any cells as input. Furthermore, a total of 14900 cells were annotated, either as relevant images, or non-relevant images. When training the aforementioned CNN classifier in a preliminary data acquisition, 14.3% of all images were discarded.

#### Graphical user interface

We developed a graphical user interface (GUI) using the Qt 5 software development tool (www.qt.io) to control all the components of the instrument. This includes settings of the camera (e.g. frame rate, gain, region of interest, output trigger and exposure time) light intensity, pressure for flow control and the mixing time. The interface can also display images as they are recorded. However, since this is computationally costly, the software can also process the images without real-time visualization. The software is able to save images according to the assigned class along with the corresponding score values. It can also be used for image processing steps such as image thresholding, contour finding, background subtraction and extracting plots of area vs shape and *x* vs *y* positions of the contour.

#### Data pre-processing

Cell images were normalized to rescale intensities to the full range representation of unsigned integers of 8 bits, *uint8* (i.e [0, 255]). The data were zero-centered using mean subtraction and the mean values were precomputed from the training dataset. Data augmentation techniques were used to increase the data pool and improve model generalizability (Bloice et al., 2019). These augmentation techniques included horizontal or vertical flips, rotations of 0,90,180 or 270 degrees and brightness and contrast changes in a range of [-10,10] % of initial value. The aforementioned techniques were applied and combined randomly for each image.

#### Deep-learning model used in strong supervised approach

A convolutional neural network (CNN) based on the ResNet18 architecture (He et al., 2016) was implemented, consisting of 18 convolutional layers (a series of convolution and identity blocks), followed by a max pooling layer, fully connected layer and a softmax activation function. A dropout layer was used before the fully connected layer, with a dropout rate of 0.9 for regularization purposes.

In a LOOCV approach, a subset of patients was used for training, while the remaining patient specimen was used as test data for validation. This results in *N*_*train*_ training iterations, with *N*_*train*_ equal to the number of patients (i.e. 9). This approach was repeated 5 times, with the test accuracy (the accuracy achieved on the patient specimen that was left out of the training process) reported throughout the paper. The data used for training were further split into a training set and a validation set at a proportion of 80% to 20%. The model was trained using a binary cross entropy loss function and optimized using a stochastic gradient descent, with a learning rate of 0.00005. A batch size of 64 was used, and the training duration was set to 250 epochs up to the point where the validation loss did not decrease for more than 10 epochs. All models were implemented in Python using Keras with a TensorFlow backend.

#### Weakly supervised approach

A convolutional auto-encoder (AE) consists of encoder and decoder structures. The encoder structure is used to transform the input into a compressed representation, termed “latent space”. The decoder structure's goal is to reconstruct the original input from the low dimensional representation. The AE used a symmetric encoder-decoder structure. The encoder consisted of 5 convolutional layers with a kernel size of 3x3 and a number of layers with values 32, 24, 16, 16 and 16. The encoder was used to represent each patient image in a latent space. The model was trained using a mean square error loss function between input and reconstructed images. The model was then optimized using *Adam* (Kingma and Ba, 2014), an adaptive learning rate optimization algorithm, with a learning rate of 0.0001, a batch size of 64, and trained for 250 epochs up to the point where the validation loss did not decrease for more than 10 epochs.

Groups of cells (N_bag_) from the same patient were defined by proximity in the latent representation, forming a BoCF of dimensions N_l_xN_bag_, where N_l_ is the latent space dimension, and the label was assigned corresponding to the disease state of the respective patient. After an average pooling operation was performed across the cells, reducing the dimensions of the BOCFs to 1xN_bag_ they were used to train a random forest classifier in a LOOCV approach. The random forest classifier is an ensemble learning method, where an ensemble of decision trees are built during training time, and the output of the random forest is the class selected by the majority of trees. In this study, the predicted class is the one with the highest mean probability estimate across all the trees in the random forest. A bag size of N_bag_ = 50 was used in all the experiments performed. The random forest classifier was implemented using the sklearn package in python. It consisted of 100 trees, with a maximum depth of 2, a maximum number of features of 10 and used the gini criterion to measure node split quality. Figure 2 illustrates the flow-chart of this approach.

#### Sample preparation for scanning electron microscope (SEM) imaging

All reagents used for SEM imaging were purchased from Sigma-Aldrich (Buchs, Switzerland). Cells in suspension were fixed in 4% Formaldehyde, washed and adhered on cleaned carbon-coated coverslips using a poly-L-lysine coating. The cell monolayer was fixed in 2.5% Glutaraldehyde, postfixed with 1% Osmiumtetroxide, treated with 1% Thiocarbohydrazide and once again with 1% Osmiumtetroxide. After incubation with 2% Uranylacetate, the monolayer was dehydrated in a series of ascending ethanol concentrations followed by stepwise immersions in Epon/Araldite resin. A resin-filled capsule was placed upside-down on top of the cells. After polymerization at 60°C the cover glass was detached and the resin block trimmed. Half of the cell was removed, then median cross-sections were cut in an ultra-microtome, and 80 nm thick sections were transferred onto silicon-wafer chips. After montage onto SEM-stubs, regions of interest were selected. Imaging was performed in a FEI Magellan 400 scanning electron microscope (FEI, Oregon, USA) at 1,8 kV and 0,8 nA by backscatter electron detection and a pixel size of 10 nm.

### Quantification and statistical analysis

#### IFC image metrics

Signal-to-noise ratio was calculated as the ratio of the average signal value $\mu_{sig}$to the standard deviation of the signal $\sigma_{sig}$, i.e.

$$SNR=\frac{\mu_{sig\;}}{\sigma_{sig\;}}$$

In addition, contrast-to-noise ratio (CNR) was defined as:

$$CNR=2\frac{{\left(\mu_{cell\;}-\mu_{bg}\right)}^{2\;}}{{\sigma_{cell}}^{2}+{\sigma_{bg}}^{2}}\text{,}$$

where $\mu_{cell\;}$and $\mu_{bg\;}$ represent the mean intensity values of the cell and background regions, respectively, and $\sigma_{cell}$ and $\sigma_{bg}$ are their corresponding standard deviations.

#### Quantification of distance between the cell predictions for the two classes

The kernel density estimation, which is a non-parametric approach for estimating the probability density function of a population, was used to model the probability density function of the predicted number of Sézary cells for both healthy and diseased patients. Afterwards, the Hellinger distance, a type of f-divergence function (one that measures the difference between two probability distributions), was used to quantify the similarity between the healthy (HD) and diseased (SS) probability functions using the formula,

$$H\left(HD,SS\right)=\frac{1}{\sqrt{2}}\sqrt{\sum_{i=1}^{k}{\left(\sqrt{hd_{i\;}}+\sqrt{ss_{i}}\right)}^{2},}$$

where $HD=\left(hd_{1},hd_{2},..,hd_{k}\right)$ and $SS=\left(ss_{1},ss_{2},..,ss_{k}\right)$ are the discrete probability density functions. The MATLAB Statistics and Machine Learning Toolbox was used for the kernel density estimation.

## Data Availability

• The raw image data reported in this study does not represent a standardized data type, being the result of a custom-built IFC device. The data reported in this paper was deposited in a public general-purpose repository. The DOI is listed in the key resources table. • This paper does not report original code. Deep learning models and machine learning models reported in this work used standard libraries and scripts that are publicly available in Keras (Chollet, 2018), TensorFlow (Abadi et al., 2016) and scikit-learn (Pedregosa et al., 2011) and listed in the key resources table. • Any additional information required to reanalyze the data reported in this paper is available from the lead contact upon request.

## Supplemental information

Document S1. Figures S1 and S2

Document S2. Article plus supplemental information
