# Supplementary material for: A weakly supervised deep learning approach for label-free imaging flow-cytometry-based blood diagnostics
Source: Cell Rep Methods. 2021 Oct 25;1(6):100094. doi: 10.1016/j.crmeth.2021.100094 (PMC9017143; doi:10.1016/j.crmeth.2021.100094)
Supplement: Document S1. Figures S1 and S2 [file mmc1.pdf]

Cell Reports Methods, Volume *1*

Supplemental information

A weakly supervised deep learning approach for label-free imaging flow-cytometry-based blood diagnostics

Corin F. Otesteanu, Martina Ugrinic, Gregor Holzner, Yun-Tsan Chang, Christina Fassnacht, Emmanuella Guenova, Stavros Stavrakis, Andrew deMello, and Manfred Claassen

Supplementary Figures

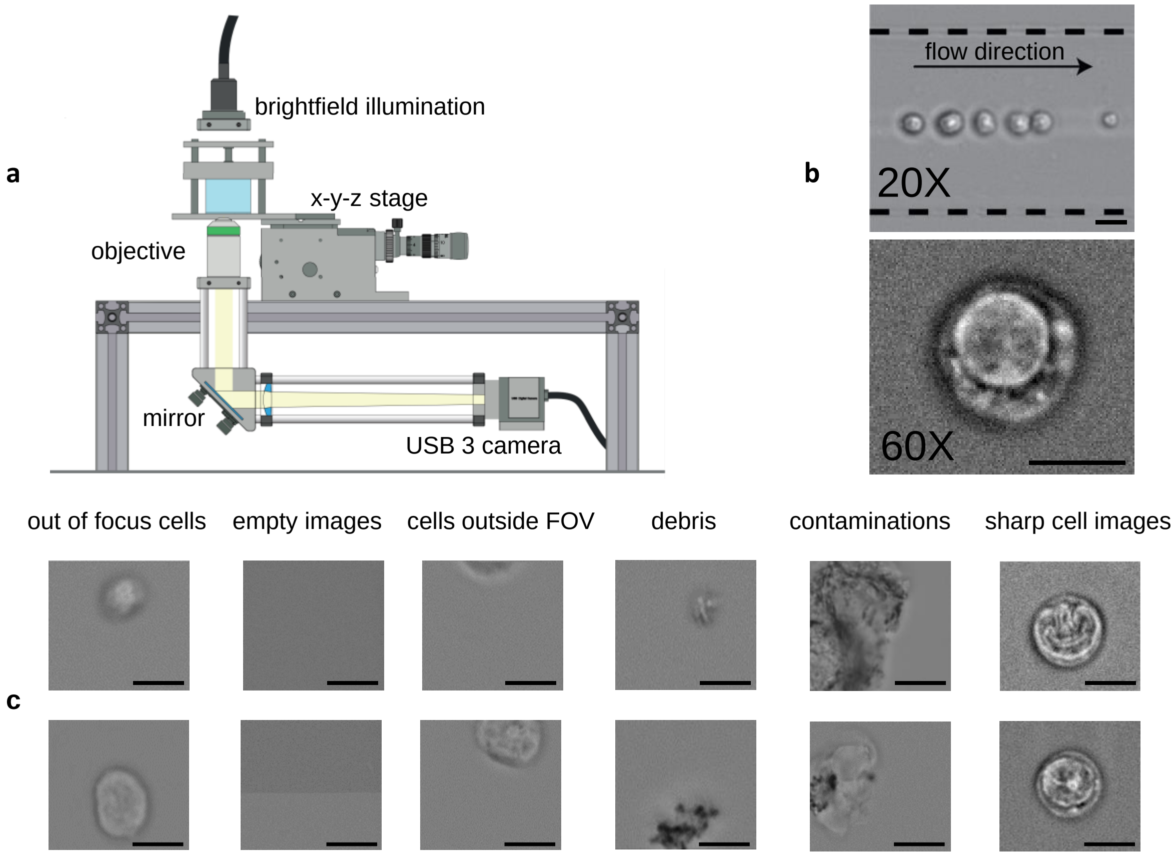

Figure S1. Schematic of the working principle of the bright field imaging flow cytometer with exemplar images. Related to Figure 1. (a) Optical system used to acquire high-resolution cell images in flow via bright field illumination. (b) Representative images of focused flowing cells at 20x and 60x magnification. (c) Cells that are out of focus or partially/fully outside the field of view can be acquired since the cells are flowing at high linear velocities within the microfluidic channel. Cellular debris and contaminants can also be present due to sample handling and preparation procedures. Only sharp cell images are used for classification. All scale bars are 5 µm.

`

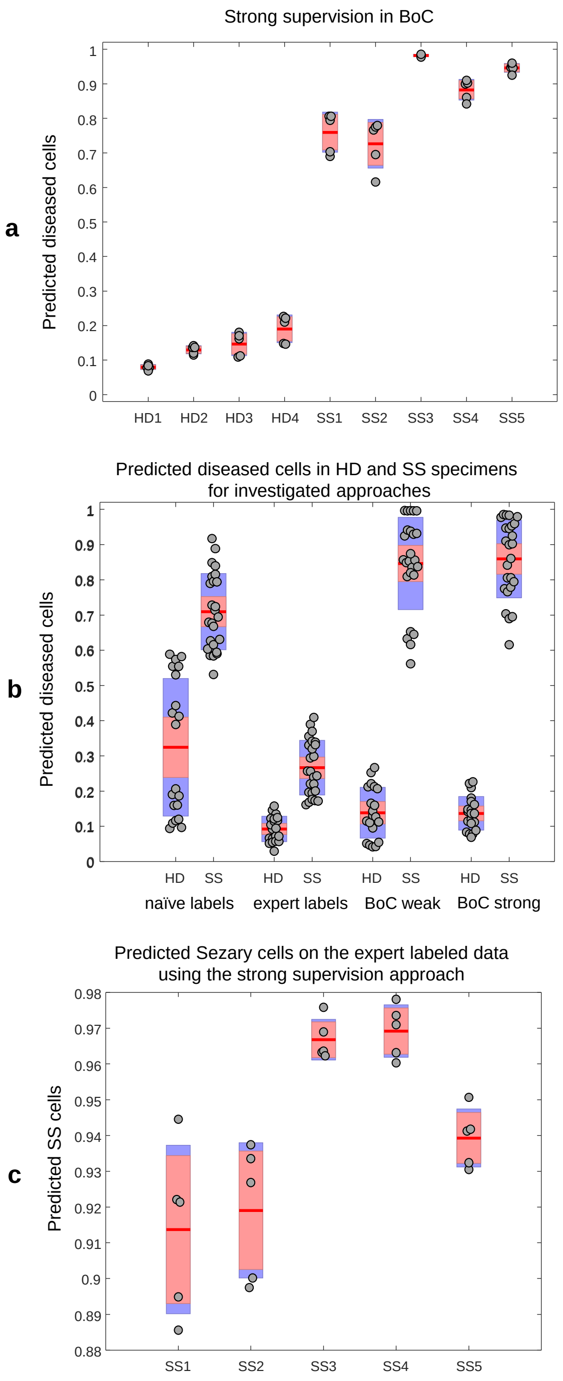

Figure S2. Strong supervision results using the BoC model. Related to Figure 3. Predicted percentage of cells with atypical (Sézary) morphology in the blood of healthy and diseased specimens using (a) the strong supervision in a bag of cell approach and (b) group-wise healthy donor and Sézary patient predictions of all the approaches investigated. (c) Classification accuracy for cells with atypical (Sézary) morphology on the expert labeled Sézary cells dataset. Evaluation was made only based on the manually annotated Sézary cell images.
